# Potential Adverse Outcomes of Shared Decision Making about Palliative Cancer Treatment: A Secondary Analysis of a Randomized Trial

**DOI:** 10.1177/0272989X231208448

**Published:** 2023-11-12

**Authors:** Loïs F. van de Water, Danique W. Bos–van den Hoek, Steven C. Kuijper, Hanneke W. M. van Laarhoven, Geert-Jan Creemers, Serge E. Dohmen, Helle-Brit Fiebrich, Petronella B. Ottevanger, Dirkje W. Sommeijer, Filip Y. F. de Vos, Ellen M. A. Smets, Inge Henselmans

**Affiliations:** Department of Medical Psychology, Amsterdam UMC location University of Amsterdam, Amsterdam, the Netherlands; Department of Medical Oncology, Amsterdam UMC location University of Amsterdam, Amsterdam, the Netherlands; Amsterdam Public Health, Quality of Care, Amsterdam, the Netherlands; Cancer Center Amsterdam, Cancer Treatment and Quality of Life, Amsterdam, the Netherlands; Department of Medical Psychology, Amsterdam UMC location University of Amsterdam, Amsterdam, the Netherlands; Amsterdam Public Health, Quality of Care, Amsterdam, the Netherlands; Cancer Center Amsterdam, Cancer Treatment and Quality of Life, Amsterdam, the Netherlands; Department of Medical Oncology, Amsterdam UMC location University of Amsterdam, Amsterdam, the Netherlands; Cancer Center Amsterdam, Cancer Treatment and Quality of Life, Amsterdam, the Netherlands; Department of Medical Oncology, Amsterdam UMC location University of Amsterdam, Amsterdam, the Netherlands; Cancer Center Amsterdam, Cancer Treatment and Quality of Life, Amsterdam, the Netherlands; Department of Medical Oncology, Catharina Ziekenhuis, Eindhoven, The Netherlands; Department of Medical Oncology, BovenIJ, Amsterdam, The Netherlands; Department of Medical Oncology, Isala Klinieken, Zwolle, The Netherlands; Department of Medical Oncology, Radboud University Medical Center, Radboud University, Nijmegen, The Netherlands; Department of Medical Oncology, Flevoziekenhuis, Almere, The Netherlands; Department of Medical Oncology, University Medical Center Utrecht, Utrecht University, Utrecht, The Netherlands; Department of Medical Psychology, Amsterdam UMC location University of Amsterdam, Amsterdam, the Netherlands; Amsterdam Public Health, Quality of Care, Amsterdam, the Netherlands; Cancer Center Amsterdam, Cancer Treatment and Quality of Life, Amsterdam, the Netherlands; Department of Medical Psychology, Amsterdam UMC location University of Amsterdam, Amsterdam, the Netherlands; Cancer Center Amsterdam, Cancer Treatment and Quality of Life, Amsterdam, the Netherlands

**Keywords:** shared decision making, advanced cancer, adverse outcomes, multivariate analysis

## Abstract

**Background:**

While shared decision making (SDM) is advocated for ethical reasons and beneficial outcomes, SDM might also negatively affect patients with incurable cancer. The current study explored whether SDM, and an oncologist training in SDM, are associated with adverse outcomes (i.e., patient anxiety, tension, helplessness/hopelessness, decisional uncertainty, and reduced fighting spirit).

**Design:**

A secondary analysis of a randomized clinical trial investigating the effects of SDM interventions in the context of advanced cancer. The relations between observed SDM (OPTION12), specific SDM elements (4SDM), oncologist SDM training, and adverse outcomes were analyzed. We modeled adverse outcomes as a multivariate phenomenon, followed by univariate regressions if significant.

**Results:**

In total, 194 patients consulted by 31 oncologists were included. In a multivariate analysis, observed SDM and adverse outcomes were significantly related. More specifically, more observed SDM in the consultation was related to patients reporting more tension (*P* = 0.002) and more decisional uncertainty (*P* = 0.004) at 1 wk after the consultation. The SDM element “informing about the options” was especially found to be related to adverse outcomes, specifically to more helplessness/hopelessness (*P* = 0.002) and more tension (*P* = 0.016) at 1 wk after the consultation. Whether the patient consulted an oncologist who had received SDM training or not was not significantly related to adverse outcomes. No relations with long-term adverse outcomes were found.

**Conclusions:**

It is important for oncologists to realize that for some patients, SDM may temporarily be associated with negative emotions. Further research is needed to untangle which, when, and how adverse outcomes might occur and whether and how burden may be minimized for patients.

**Highlights:**

- Observed shared decision making was related to more tension and uncertainty postconsultation in advanced cancer patients
- However, training oncologists in SDM did not affect adverse outcomes.
- Further research is needed to untangle which, when, and how adverse outcomes might occur and how burden may be minimized

Patients with incurable cancer need to make complex treatment decisions, since treatment options often have no clear benefit-harm ratio: the outcomes are uncertain, the survival benefit is often limited, and the burden of treatment can be high. As a result, patients’ preferences are decisive in determining the best treatment strategy. Such preference-sensitive treatment selection requires shared decision making (SDM).^1,2^ SDM is a way of involving both health care professionals’ experience and expertise as well as patients’ values and preferences in the treatment decision-making process^1,3^ and is considered the pinnacle of patient-centered care.^4^ The value of SDM originated mainly from ideological and bioethical bases,^5,6^ with patient autonomy being a central ethical imperative.^1,7^ Importantly, most patients want to be involved in cancer treatment decision-making process,^8,9^ especially during the palliative phase.^10,11^

Aside from its ethical imperative, SDM has been advocated because of evidence for its beneficial effects, such as on patients’ satisfaction with communication, well-being, and quality of life.^12–14^ Nevertheless, SDM might also inflict burden on patients,^15,16^ which may conflict with other ethical principles of care, such as nonmaleficence and beneficence.^5,17^ SDM implies partly attributing responsibility for decision making to people in a vulnerable position, which might empower yet also weigh down on them.^7,18–20^ Also, for effective SDM, transparent and elaborate communication is required about prognosis as well as the benefits and harms of different treatment options. This information might be confronting for patients, especially in palliative cancer care. Furthermore, such information is often uncertain and not evidence based and may trigger uncertainty in patients relating to probabilities of individual responses to treatment^18^ and the effect of the illness or treatment on one’s personal life.^21^ All of this may complicate health care professionals’ information provision and patients’ comprehension and considerations.^7,18,20^

While the potential burden of SDM is acknowledged in the literature, little evidence exists to explain or support this phenomenon. There is tentative evidence that some patients may experience adverse outcomes after engaging in SDM, such as anxiety, dissatisfaction with the decision, and increased decisional conflict,^13,22^ which may coincide with feelings of helplessness or hopelessness or a lower fighting spirit.^23^ Furthermore, confronting information about benefits and harms might trigger emotional stress or anxiety.^24–26^ Possibly, the experienced burden of SDM differs by how SDM is performed (e.g., how well the performed decision-making process fits the needs and wants of a specific patient)^27–29^ and which elements of SDM are predominant. For example, when precise information giving about pros and cons is not combined with sufficiently supporting patients in the construction of treatment preferences, they might end up feeling confused or lost. If we better understand potential adverse outcomes of SDM, physicians could anticipate on these during consultations and try to prevent or lower the possible burden for patients.^30^

The current study aims to explore whether SDM is associated with adverse outcomes, based on data of a previous study, the CHOICE-trial.^31,32^ In this randomized trial, it was demonstrated that a training in SDM had a large positive effect on observed and patient-reported SDM in oncological consultations while a patient conversation aid did not. By a secondary analysis of the CHOICE data, we investigate whether SDM, and an oncologist training in SDM, are associated with adverse outcomes in the context of advanced cancer. Specifically, we want to examine whether SDM is associated with patients’ anxiety, helplessness/hopelessness, uncertainty, and fighting spirit. In addition, we will explore how specific elements of SDM (i.e., raising choice awareness, informing about options, and preference construction and their combination) are related to these outcomes. We will explore associations on both the short term and the long term. Lastly, we examine the potential moderating effect of patients’ decisional role preferences and of the type of decision, that is, about either the start or the continuation of therapy, as both might define the burden of SDM.^27–29^

## Methods

### CHOICE Trial

This article describes a secondary analysis of data collected for the CHOICE trial (CHOosing treatment together In Cancer at the End of life; Netherlands Trial Registry NTR5489). A multicenter, randomized controlled design with 4 parallel arms was adopted to examine the independent and combined effect of an oncologist training and a patient communication aid on observed SDM about palliative cancer treatment in clinical consultations.^32,33^ Detailed information about the CHOICE trial can be found in the trial protocol.^31^ The protocol was approved by the Medical Ethical Committee of the coordinating center (Academic Medical Center, University of Amsterdam; NL 48722.018.15; METC-2015-149) and local feasibility by all participating centers. All procedures were conducted in line with the Declaration of Helsinki. All participants signed informed consent.

### Recruitment and Sample

Medical oncologists and medical oncologists in training, who were recruited through existing networks, were eligible when treating patients with metastatic or inoperable tumors. Patients scheduled for an initial or evaluative consultation to discuss the start, (dis)continuation, or adjustment of palliative systemic treatment in 7 Dutch hospitals were invited for participation. Eligible patients were diagnosed with metastatic or inoperable tumors for which the indicated median life expectancy without disease-targeted treatment was less than 12 mo and palliative systemic treatment would not offer a median survival benefit of more than 6 mo.

### Data Collection

Oncologists and patients with advanced cancer were randomized to receive an SDM skills training and a patient communication aid, respectively. Consultations about advanced cancer treatment decisions were audio recorded and assessed by trained and blinded observers. Also, patients filled out questionnaires at baseline (T0), in the waiting room (T1), and at 1 wk (T2), 3 mo (T3), and 6 mo (T4) after the consultation. Data collection took place between February 2016 and June 2018.

### Measurements

#### Sample characteristics

Patients reported their age, gender, educational level (low, elementary to low vocational education; medium, up until medium-level vocational education; high, high vocational or academic education), nationality, and preferred decision-making role by questionnaire. In the local case report forms, the patient’s tumor type and the type of consultation (first/evaluative) were registered; the results of the (positron emission tomography [PET])computed tomography (CT) scan in case of evaluative consultations, patients’ World Health Organization performance status at the time of the consultation (0–4), and the line of therapy that was discussed during the consultation were extracted from the medical record.

#### Outcome variables

For the current analysis, the primary outcomes were the adverse outcomes as self-reported by patients shortly after the consultation (T2): anxiety, tension, loss of fighting spirit, helplessness/hopelessness, and decisional uncertainty. *Anxiety* was measured with the 6-item short version of the state scale of the Spielberger State and Trait Anxiety Inventory (STAI-6),^34^ measuring state anxiety on a 4-point Likert-type scale (1 = *not at all* to 4 = *very much so*). Another 1-item visual analogue scale measurement of anxiety was performed to assess (momentary) *tension. Loss of fighting spirit* was measured with the 4-item subscale of the mini-Mental Adjustment to Cancer subscale (mini-MAC),^35^ and *helplessness/hopelessness* was measured with the 6-item subscale of the Mental Adjustment to Cancer subscale (MAC).^36,37^ The MAC was developed to assess specific responses to cancer, and all items could be rated on a 4-point Likert-type scale (1 = *definitely not applicable* to 4 = *definitely applicable*). Lastly, *decisional uncertainty* was measured with the 3-item uncertainty subscale of the Decisional Conflict Scale,^38^ measuring the uncertainty about choosing among alternatives (such as “I am clear about the best choice from me,”“This decision is easy for me to make”); items could be rated on a 5-point Likert-type scale (1 = *completely disagree* to 5 = *completely agree*).

To explore whether SDM and adverse outcomes were related after a longer period of time, measurements of adverse outcomes at 3 (T3) and 6 mo (T4) after the consultation were used. For decisional uncertainty, no long-term measurements were available.

#### Independent variables

Observed SDM was assessed from audio-recorded consultations using the Observing Patient Involvement in Decision-Making scale (OPTION12), which aims to assess the extent to which health care professionals involve patients in the decision-making process.^39,40^ Twelve items were rated on a 5-point Likert-type scale (0 = *behavior not observed* to 4 = *behavior is observed and executed to a high standard*), and the summed score was transformed to reflect a total out of 100.

The effects of potentially burdensome elements of SDM were analyzed separately. SDM elements were defined using the separate stages of Stiggelbout et al.’s model of SDM (stage 1, “setting the SDM agenda”; stage 2, “informing about the options”; and stage 3, “exploring patient values and preferences”^1^) as assessed with the 4-stage model of SDM (4SDM).^33^ The 4SDM contains 2 items per stage, which were coded on a 4-point scale (0 = *not observed* to 3 = *observed and of high quality*). Stage 4, “reaching a joint decision,” was not included in the current study.

Two trained and blinded assessors independently rated the consultation using both scales (OPTION12 and 4SDM) after training and calibration. The intraclass correlation was strong (>0.80) and the average weighted kappa sufficient was >0.60 for OPTION12 (0.62) and was almost sufficient for 4SDM (0.57).^32^

The second independent variable was the intervention condition of the oncologist (i.e., whether the patient consulted a trained or untrained oncologist). The patient communication aid condition was ignored for this secondary analysis, as the observed SDM behavior of oncologists was not shown to be affected by the aid.^32^

#### Moderating variables

Patients’ preferred role in decision making was measured using the Control Preferences Scale (T0, a 1-item measure with 5 different treatment decision-making roles^41^). The items were rearranged from A/B/C/D/E to AB/C/DE to reflect an active, shared, or passive role.^42^

Type of consultation was categorized as 1) initial consultation (consultation to discuss the start of [a new line of] treatment) or 2) evaluative consultation (consultation to discuss the [dis]continuation or adjustment of treatment on the basis of CT or PET-CT evaluation).

### Statistical Analyses

To investigate the relation between SDM and adverse outcomes as well as the effect of the SDM training on adverse outcomes, we modeled adverse outcomes as a multivariate (i.e., multiple outcomes) phenomenon, because the individual adverse outcomes correlated (*r* = 0.20–0.81; see Appendix A, Table A.1). In all multivariate regression analyses, only significant multivariate effects were followed up with univariate regressions to interpret the direction of the effect. For all analyses, 2-sided *P* values ≤0.05 were considered significant. Furthermore, the nesting structure of patients treated by oncologists was not accounted for since the intraclass correlations were very small (>0.02; see Appendix A, Table A.2). Missing data were imputed with a random forest imputation using the R-package missForest, and all analyses were performed in R version 4.0.3 and Rstudio.^43,44^

#### Shared decision making

To model the relation between observed SDM and adverse outcomes, we fit a multivariate model that included all adverse outcomes at the first follow-up measurement (T2) as outcome variables (anxiety, tension, helplessness/hopelessness, loss of fighting spirit, uncertainty) and observed SDM as well as baseline scores of all adverse outcomes as independent variables. Including baseline adverse outcomes (T0) in the model allowed us to control for baseline differences between patients. For tension, the measurement shortly before the consultation in the waiting room (T1) was considered the baseline. For uncertainty, no preconsultation baseline measurements were available.

To test if the relationship between SDM and adverse outcomes was moderated by preferred role in decision making and type of conversation, we also tested for multivariate interactions. In univariate analyses of SDM and each of the separate adverse outcomes, we controlled only for the corresponding baseline adverse outcome to reduce multicollinearity.

A similar modeling approach was used to investigate the relationship between the separate SDM elements and adverse outcomes. We fit a multivariate model for adverse outcomes at follow-up using the individual elements of SDM and baseline adverse outcomes, after which we also tested for interactions between stage 1 (agenda setting) and 3 (exploration) and between stage 2 (information giving) and 3 (exploration). Again, both multivariate and univariate analyses were controlled for baseline differences and moderating variables.

#### Intervention condition (oncologist training)

The effect of oncologist training on adverse outcomes was multivariately modeled using a dichotomous variable representing whether the oncologist was trained or not as an independent variable. If the effect of oncologist training was significant, we controlled for baseline and background variables (see the “Sample Characteristics” section) that were significantly different between patients consulted by a trained or untrained oncologist.

#### Long-term outcomes

For testing the relationship between observed SDM and long-term adverse outcomes (T3, 3 mo; T4, 6 mo), and the effect of oncologist training on long-term adverse outcomes, identical models were used as those used for analysis of the measurements shortly after the consultation T2, see Statistical analyses- Shared decision making and Statistical analyses- Intervention condition (oncologist training)). As for decisional uncertainty, no measurements at T3 nor T4 were available; this outcome variable was excluded from the models. To ensure sufficient statistical power at T3 and T4, analyses were performed only if the dropout percentage compared with T2 was small enough for imputation (<50%).

## Results

A total of 194 patients, consulted by 31 oncologists from 7 different hospitals, participated (Table 1). Audio recordings of consultations were collected for 187 patients (96%), on average 6 ± 3.64 mo after the training of oncologists (range: 0–15 mo); 168 patients (87%) returned the postconsultation questionnaire. Means and standard deviations of adverse outcomes at different time points are displayed in Appendix B. We were unable to perform analyses of adverse outcomes at 6 mo after the consultation (T4) due to the small number of participants remaining in the sample at this point in time (sample size: *n* = 84, 43% of *N* = 194 at T2).

**Table 1:** Participant Characteristics at T2 (Total Sample)

	Total	Oncologist SDM Training	
	(*N* = 194)	No (*n* = 99)	Yes (*n* = 95)
Patient characteristics			
Age, M (SD)	63.6 (11.2)	62.9 (11.1)	64.4 (11.3)
Sex, % (*n*) male	51.0 (99)	48.5 (48)	53.7 (51)
Educational level,^a^ % (*n*)			
Low	34.8 (62)	31.9 (30)	38.1 (32)
Medium	25.8 (46)	28.7 (27)	22.6 (19)
High	39.3 (70)	39.4 (37)	39.3 (33)
Dutch nationality,^a^ % (*n*) versus other	98.3 (176)	98.9 (93)	97.6 (83)
Preferred decision-making role,^a^ % (*n*)			
Passive	11.0 (18)	11.8 (10)	10.3 (8)
Shared	65.0 (106)	68.2 (58)	61.5 (48)
Active	23.9 (39)	20.0 (17)	28.2 (22)
Disease characteristics			
Tumor type,^b^ % (*n*)			
Pancreatic	20.6 (40)	18.2 (18)	23.2 (22)
Esophagogastric	20.6 (40)	10.1 (10)	31.6 (30)
Gynecological	10.8 (21)	13.1 (13)	8.4 (8)
Other gastrointestinal	10.8 (21)	7.1 (7)	14.7 (14)
Colorectal	9.8 (19)	8.1 (8)	11.6 (11)
Urogenital	7.2 (14)	14.1 (14)	0
Mamma	5.7 (11)	11.1 (11)	0
Melanoma	5.2 (10)	8.1 (8)	2.1 (2)
Other	9.3 (18)	10.1 (10)	8.4 (8)
Results (PET)CT,^a,c^ % (*n*)			
Stable or response	68.6 (81)	74.6 (50)	60.8 (31)
Progression	28.0 (33)	22.4 (15)	35.3 (18)
NA (no CT made)	3.4 (4)	3.0 (2)	3.9 (2)
World Health Organization status, % (n)			
0–1	81.5 (145)	77.3 (68)	85.6 (77)
2–4	18.5 (33)	22.7 (20)	14.4 (13)
Consultation characteristics			
Evaluative consult, % (*n*), versus first	61.3 (119)	67.7 (67)	54.7 (52)
Line of treatment discussed,^a,d^ % (*n*)			
1	56.0 (108)	48.5 (48)	63.8 (60)
≥2	44.0 (85)	51.5 (51)	36.2 (34)

CT, computed tomography; NA, not applicable; PET, positron emission tomography; SDM, shared decision making.

a Missing: educational level *n* = 16; Dutch nationality *n* = 15; preferred decision-making role *n* = 31; results of (PET)CT and line of treatment discussed *n* = 1.

b Significant difference across conditions, *P* < 0.01.

c Results (PET)CT were reported only in case of a evaluative type of consultation; see this characteristic for total *n*.

d Significant difference across conditions, *P* < 0.05.

### SDM and Adverse Outcomes

At 1 wk after the consultation, observed SDM as measured with the OPTION12 was significantly related to the adverse outcomes in multivariate analysis corrected for baseline variables (*P* > 0.001; see Table 2, T2). Univariate analyses of observed SDM on the separate outcomes demonstrated significantly higher tension (*P* = 0.002) and higher uncertainty (*P* = 0.004, no baseline control available) when more SDM was observed (see Table 3).

**Table 2:** Multivariate Effects of Overall Observed SDM on Adverse Outcomes at T2 (1 wk) and T3 (3 mo), When Corrected for Baseline Variables^a^

Time Point	T2 (1 wk)		T3 (3 mo)	
Predictor	Pillai	Pr(>F)	Pillai	Pr(>F)
**Model 1: SDM (OPTION12) + baseline variables**				
SDM (OPTION12)	0.183	<0.001^b^	0.042	0.286
Baseline anxiety (STAI)	0.515	<0.001	0.385	<0.001
Baseline loss of fighting spirit	0.578	<0.001	0.490	<0.001
Baseline helplessness/hopelessness	0.285	<0.001	0.184	<0.001
Baseline tension (VAS)	0.056	0.057	0.056	0.150
**Model 2: SDM (OPTION12) + baseline variables + preferred role interaction**				
SDM (OPTION12)	0.188	<0.001	0.042	0.298
Preferred role	0.074	0.180	0.098	0.170
Baseline anxiety (STAI)	0.521	<0.001	0.370	<0.001
Baseline loss of fighting spirit	0.572	<0.001	0.493	<0.001
Baseline helplessness/hopelessness	0.286	<0.001	0.190	<0.001
Baseline tension (VAS)	0.054	0.076	0.048	0.234
Interaction preferred role x SDM (OPTION12)	0.078	0.150^c^	0.053	0.625
**Model 3: SDM (OPTION12) + baseline variables + consultation type interaction**				
SDM (OPTION12)	0.184	<0.001	0.043	0.280
Consultation type	0.068	0.024	0.027	0.528
Baseline anxiety (STAI)	0.514	<0.001	0.399	<0.001
Baseline loss of fighting spirit	0.578	<0.001	0.492	<0.001
Baseline helplessness/hopelessness	0.286	<0.001	0.189	<0.001
Baseline tension (VAS)	0.060	0.043	0.051	0.196
Interaction consultation type × SDM (OPTION12)	0.022	0.525^c^	0.045	0.249

SDM, shared decision making; STAI, State and Trait Anxiety Inventory; VAS, visual analog scale.

a Results are shown separately for each model (with and without interactions).

b Significant univariate effects of overall observed SDM on specific adverse outcomes at *P* < 0.05.

c Indicates no significant multivariate interaction effect.

**Table 3:** Univariate Models and Effects of Overall Observed SDM on the Separate Adverse Outcomes at T2 (1 wk), when Corrected for Baseline Variables

Outcome	Predictor	Estimate	*P* Value
Anxiety (STAI)	(Intercept)	0.45	<0.001
	SDM (OPTION12)	0.00	0.33
	Baseline anxiety (STAI)	0.74	<0.001
Loss of fighting spirit	(Intercept)	1.54	0.004
	SDM (OPTION12)	0.01	0.42
	Baseline loss of fighting spirit	0.82	<0.001
Helplessness/hopelessness	(Intercept)	4.24	<0.001
	SDM (OPTION12)	0.01	0.42
	Baseline helplessness/hopelessness	0.61	<0.001
Tension (VAS)	(Intercept)	1.49	0.73
	SDM (OPTION12)	0.29	0.002^a^
	Baseline tension (VAS)	0.35	<0.001
Uncertainty	(Intercept)	20.57	<0.001
	SDM (OPTION)	0.24	0.004^a^

SDM, shared decision making; STAI, State and Trait Anxiety Inventory.

a Significant univariate effects of overall observed SDM on specific adverse outcomes at *P* < 0.05.

Interactions between SDM and both preferred role (*P* = 0.064) as well as type of consultation (*P* = 0.53) did not have significant multivariate effects on adverse outcomes (Table 2, T2), meaning that the relation between SDM and outcomes did not depend on patients’ preferred role nor on the type of consultation. Hence, univariate interactions were not explored.

At 3 mo postconsultation (T3, sample size: *n* = 126, 65% of *N* = 194 at T2), observed SDM was not significantly related to adverse outcomes, corrected for baseline variables (*P* = 0.29; Table 4, T3). Adding the interactions between SDM and preferred role as well as type of consultation also did not yield significant multivariate models (see Table 4, T3).

**Table 4:** Multivariate Effects of Observed SDM in the Elements of “Setting the SDM Agenda,”“Informing about the Options,” and “Exploring Patient Values and Preferences” on Adverse Outcomes, at T2 (1 wk) and T3 (3 mo), when Corrected for Baseline Variables^a^

Time Point	T2 (1 wk)		T3 (3 mo)	
Predictor	Pillai	Pr(>F)	Pillai	Pr(>F)
**Model 1: stages 1, 2, 3 + baseline variables**				
Stage 1: Setting the SDM agenda	0.030	0.356	0.039	0.327
Stage 2: Informing about the options	0.141	<0.001^b^	0.047	0.233
Stage 3: Exploring patient values and preferences	0.014	0.760	0.047	0.229
Baseline anxiety (STAI)	0.529	<0.001	0.394	<0.001
Baseline loss of fighting spirit	0.483	<0.001	0.301	<0.001
Baseline helplessness/hopelessness	0.467	<0.001	0.425	<0.001
Baseline tension (VAS)	0.052	0.080	0.063	0.109
**Model 2: stages 1, 2, 3 + baseline variables + stage 1 × stage 3 interaction**				
Stage 1: Setting the SDM agenda	0.030	0.349	0.039	0.330
Stage 2: Informing about the options	0.119	<0.001^b^	0.041	0.305
Stage 3: Exploring patient values and preferences	0.043	0.155	0.053	0.177
Baseline anxiety (STAI)	0.535	<0.001	0.395	<0.001
Baseline loss of fighting spirit	0.483	<0.001	0.303	<0.001
Baseline helplessness/hopelessness	0.467	<0.001	0.427	<0.001
Baseline tension (VAS)	0.052	0.081	0.063	0.112
Interaction stage 1 × stage 3	0.026	0.430	0.017	0.743
**Model 3: stages 1, 2, 3 + baseline variables + stage 2 × stage 3 interaction**				
Stage 1: Setting the SDM agenda	0.030	0.351	0.039	0.331
Stage 2: Informing about the options	0.142	<0.001^b^	0.047	0.236
Stage 3: Exploring patient values and preferences	0.014	0.759	0.047	0.232
Baseline anxiety (STAI)	0.532	<0.001	0.395	<0.001
Baseline loss of fighting spirit	0.483	<0.001	0.302	<0.001
Baseline helplessness/hopelessness	0.467	<0.001	0.425	<0.001
Baseline tension (VAS)	0.052	0.081	0.063	0.111
Interaction stage 2 × stage 3	0.025	0.466	0.004	0.976

a Results are shown separately for each model (with and without interactions).

b Significant univariate effects of overall observed SDM on specific adverse outcomes at *P* < 0.05.

### SDM Elements and Adverse Outcomes

At 1 wk postconsultation, the observed SDM elements “setting the SDM agenda” and “exploring patient values and preferences” were not significantly related to the adverse outcomes in the multivariate analysis corrected for baseline variables (agenda setting: *P* = 0.36, exploring: *P* = 0.76; see Table 4, T2). However, the SDM element “informing about the options” was significantly related to the adverse outcomes (*P* > 0.001; Table 4, T2). Univariate analyses showed significantly higher helplessness/hopelessness (*P* = 0.002) and higher tension (*P* = 0.016; see Table 5), both controlled for baseline variables, when more “informing about the options” was observed.

**Table 5:** Univariate Models and Effects of Observed SDM in the Elements “Setting the SDM Agenda,”“Informing about the Options,” and “Exploring Patient Values and Preferences” on the Separate Adverse Outcomes at T2 (1 wk) when Corrected for Baseline Variables

Outcome	Predictor	Estimate	*P* Value
Anxiety (STAI)	(Intercept)	0.53	<0.001
	Stage 1: Setting the SDM agenda	−0.02	0.56
	Stage 2: Informing about the options	0.01	0.67
	Stage 3: Exploring patient values and preferences	0.00	0.95
	Baseline anxiety (STAI)	0.75	<0.001
Loss of fighting spirit	(Intercept)	1.78	0.003
	Stage 1: Setting the SDM agenda	−0.11	0.36
	Stage 2: Informing about the options	0.13	0.24
	Stage 3: Exploring patient values and preferences	−0.01	0.92
	Baseline loss of fighting spirit	0.82	<0.001
Helplessness/hopelessness	(Intercept)	4.67	<0.001
	Stage 1: Setting the SDM agenda	−0.32	0.026^a^
	Stage 2: Informing about the options	0.41	0.002^b^
	Stage 3: Exploring patient values and preferences	v0.14	0.22
	Baseline helplessness/hopelessness	0.62	<0.001
Tension (VAS)	(Intercept)	9.83	0.063
	Stage 1: Setting the SDM agenda	−2.97	0.046^a^
	Stage 2: Informing about the options	3.36	0.016^b^
	Stage 3: Exploring patient values and preferences	0.42	0.71
	Baseline tension (VAS)	0.36	<0.001
Uncertainty	(Intercept)	23.04	<0.001
	Stage 1: Setting the SDM agenda	−1.41	0.30
	Stage 2: Informing about the options	2.19	0.083
	Stage 3: Exploring patient values and preferences	0.93	0.36

a Significant univariate effects, without significant multivariate effects. Following our analysis plan, these were not considered significantly related.

b Significant univariate effects of overall observed SDM on specific adverse outcomes at *P* < 0.05.

Interactions between “setting the SDM agenda” and “exploring patient values and preferences” (*P* = 0.35) and between “informing about the options” and ‘exploring patient values and preferences” (*P* = 0.94) did not significantly relate to adverse outcomes in the multivariate analysis (Table 4, T2, models 2 and 3). Hence, no univariate interaction effects were tested.

At 3 mo postconsultation, no significant multivariate relations were found between the SDM elements and adverse outcomes (agenda setting: *P* = 0.33, informing: *P* = 0.23, exploring: *P* = 0.23; Table 4, T3) nor between their interactions and adverse outcomes (see Table 4, T3).

### Oncologist Training and Adverse Outcomes

At 1 wk postconsultation, oncologist training did not have a significant multivariate effect on the adverse outcomes (*P* = 0.25). Oncologist training did not affect adverse outcomes at 3 mo either (*P* = 0.43). Hence, univariate effects were not explored.

## Discussion

In the current study, we explored the relation between SDM and potential adverse patient outcomes (i.e., anxiety, tension, loss of fighting spirit, helplessness/hopelessness, and uncertainty). We found that SDM and multivariate short-term adverse outcomes were significantly related. More specifically, more SDM in the consultation was related to more tension and decisional uncertainty after the consultation. “Informing about the options” was the only SDM element found to be significantly related to adverse outcomes (i.e., to more helplessness/hopelessness and tension) after the consultation. These results imply SDM may be associated with negative emotions for some patients. Whether the patient consulted with an SDM-trained oncologist or not did not affect adverse outcomes. In addition, we did not find any relation with long-term adverse outcomes, suggesting that the found associations are transient.

Our finding that SDM might not only be related to beneficial but also to adverse outcomes is in line with speculations and findings in the literature.^13,22^ While these outcomes may be unwanted, Politi et al.^18^ suggested that some anxiety and uncertainty cannot be avoided if we want patients to truly understand the benefits as well as the harms of treatment options and assign them partial responsibility, both needed to engage in decision making. However, because we did not find any relations between SDM and adverse outcomes in the long term, we assume these emotions to be transitory. Yet, it is important for daily executors of SDM to realize that patients might temporarily feel more tense and more uncertain in relation to the decision. Future research should explore how oncologists should deal with this complicated balance of exposing patients to challenging information and responsibility while at the same time trying to reduce potential burden as much as possible. Possible solutions may be for oncologists to tailor their information provision or communications styles or offer support with making the decision,^45^ for example, by paying more attention to exploring patients’ uncertainties, which seems not to be routine yet for health care professionals.^46,47^ This might in turn help them to determine how to tailor their information to the needs and wants of the patient.^46^ Also, an interprofessional approach to SDM, in which other health care professionals aside from the oncologist are involved in SDM,^48^ may help to identify adverse outcomes and support patients in coping with them.

It may be important to further reflect on the concept of “adverse outcomes.” In this study, we focused on outcomes related to patients’ emotional status, that is (a combination of) patients’ anxiety, tension, reduced fighting spirit, helplessness/hopelessness and decisional uncertainty. However, there may be various other outcomes playing a role in the potential burden of SDM for patients, for example, cognitive factors such as dissatisfaction with the decision and decisional conflict or relational outcomes between oncologist and patient, such as a potential decrease in patients’ trust in the oncologist, perceived empathy, or feeling of support by the oncologist. In addition, it is still unknown how potential adverse outcomes relate to and interact with each other. It might, for instance, be that decisional uncertainty would mediate the relationship between SDM and tension, which should be explored in further research. Lastly, we have included a reduced fighting spirit as one of the “adverse outcomes,” yet one could similarly argue that reduced fighting spirit, perhaps accompanied by a more accepting stance, actually reflects an adaptive response when facing incurable cancer. In line, a recent study showed that patients with low fighting spirit were more likely to have prognostic perceptions similar to that of their oncologist.^49^

Interestingly, our study showed no influence of patient role preferences on the relation between SDM and adverse outcomes; that is, whether a patient prefers an active v. a rather passive role in decision making does not influence experienced SDM burden as much as we expected. Nonetheless, it is of great importance that oncologists take into account their patients’ needs and wants when making a decision, also in regard to their preferred decisional role. Inherent to the principles of SDM, SDM should be regarded as means to an end and not be executed at any cost, as this would contradict the idea that patients’ preferences are accounted for.^1,3^

When considering the relations between separate elements of SDM and adverse outcomes, adequately informing patients on the available options and their pros and cons was found to be related to patients experiencing more tension and helplessness/hopefulness shortly after the consultation. It could be that the potentially challenging information on life expectancy, which is uncertain and in the case of advanced cancer often limited, plays a key role in this relationship. In fact, it might not be surprising that patients who have just learned that treatment can prolong their life with only an extra few months feel hopeless or tense. Especially since earlier research suggests that many patients with advanced cancer are not aware that chemotherapy is not intended to cure their cancer.^50^ However, the literature is not unequivocal on the effects of disclosure of prognostic information.^51^ Hagerty et al.^52^ reported mixed feelings among patients about the hope that exact prognosis information would give them, while Mack et al.^53^ found that disclosure of prognostic information can even support hope in patients, even when prognosis is poor. Yet, not all patients want to know their life expectancy; in the setting of advanced cancer, this can reach up to 30% of patients.^54^ Especially when, regardless of their patients’ information preference not to know or not to know in detail, doctors provide extensive information on this topic, it might lead to burden. Aside from prognosis, it might be that other aspects of information on the options and pros and cons cause patients to feel more tense and more helpless/hopeless, such as information on the side effects or the frequent hospital visits associated with treatment. It might, for instance, be that patients beforehand have unrealistic expectations of the available treatment options and their outcomes, possibly leading to a decrease in hope and increase in stress after having heard the truth about what the oncologist has to offer them. Related to our finding that more observed SDM is associated with more tension and uncertainty, it could be that patients get slightly overwhelmed by the information they receive about the treatment options. However, the fact that we did not find any significant relation to long-term adverse outcomes suggests that the burden might be temporary for patients. The increased negative emotions might wear out over time and possibly even be replaced by positive outcomes in the long-term. In addition, many other events may take place, and decisions are made in the time span of being sick, which might even invoke new emotions in patients. Future research should look into other outcomes and more long-term outcomes, such as satisfaction and the decisional regret, of patients who were involved in decision making.

Although we did find observed SDM to be related to adverse outcomes, we did not find a significant effect of SDM training on adverse outcomes in the short or long term. This means that we did not find either more or less adverse outcomes in the training condition compared with the control condition. Despite the fact that the training was not targeted at preventing adverse outcomes in patients, possibly trained oncologists still managed to perform SDM in a way that did not raise adverse outcomes as opposed to SDM performed by untrained oncologists. On the other hand, it might be that trained oncologists performed SDM more standardly, independent of patients’ context and circumstances, and—related—their emotional well-being. Either way, future SDM training programs should inform health care professionals that SDM might relate to negative emotions and provide them with tools on how to offer support in these situations. Further research should explore how patients experience burden and what might help them to reduce it to a minimum.

### Limitations

First, our finding that decisional uncertainty might be an adverse outcome of SDM should be interpreted with some caution, as in the current study we could not take into account the level of uncertainty that patients experienced at baseline. It might also be the case that uncertain or doubting patients would induce SDM behavior in oncologists. For example, if patients seem uncertain or hesitant about a treatment decision, oncologists may explain treatment options more elaborately or engage in preference construction. Second, despite the baseline controls performed for the other adverse outcomes, causality can also not be claimed for the other relationships found, due to the cross-sectional design of this study. The fact that a randomly assigned and highly effective oncologist training in SDM did not significantly relate to adverse outcomes might further substantiate that the reported relation between observed SDM and adverse outcomes is not necessarily, or purely, causal. Third, the follow-up measurement took place not directly but 1 wk after the consultation, possible influencing the outcomes. Lastly, it is important to bear in mind that our results apply only to the specific setting of patients with advanced cancer, defined as having a median life expectancy <12 mo and an expected median survival benefit of <6 mo due to treatment. Further research should determine how these results would translate into other cancer settings.

## Conclusion

The current study shows a relationship between SDM and adverse outcomes in patients with advanced cancer; specifically, an increase in tension and uncertainty was observed to coincide with more SDM. It is important for oncologists to realize that short-term negative emotions might occur for patients when engaging in SDM, for which oncologists could be equipped through future SDM training programs addressing this issue. The current oncologist SDM training did not affect adverse outcomes. Although our results do not show what the exact cause of adverse outcomes might be, they do show that the element of informing patients might specifically be associated with short-term adverse outcomes (i.e., patients might temporarily feel more tense and more helpless or hopeless when adequate information is given in order to decide on treatment). However, further research is needed to untangle exactly which, when, and how adverse outcomes of SDM might occur, how patients experience these, and how burden might be minimized.

## Supplemental Material

sj-docx-1-mdm-10.1177_0272989X231208448 – Supplemental material for Potential Adverse Outcomes of Shared Decision Making about Palliative Cancer Treatment: A Secondary Analysis of a Randomized Trial Click here for additional data file. Supplemental material, sj-docx-1-mdm-10.1177_0272989X231208448 for Potential Adverse Outcomes of Shared Decision Making about Palliative Cancer Treatment: A Secondary Analysis of a Randomized Trial by Loïs F. van de Water, Danique W. Bos–van den Hoek, Steven C. Kuijper, Hanneke W. M. van Laarhoven, Geert-Jan Creemers, Serge E. Dohmen, Helle-Brit Fiebrich, Petronella B. Ottevanger, Dirkje W. Sommeijer, Filip Y. F. de Vos, Ellen M. A. Smets and Inge Henselmans in Medical Decision Making
