# Supplementary material for: Potential Adverse Outcomes of Shared Decision Making about Palliative Cancer Treatment: A Secondary Analysis of a Randomized Trial
Source: Med Decis Making. 2023 Nov 12;44(1):89–101. doi: 10.1177/0272989X231208448 (PMC10712204; doi:10.1177/0272989X231208448)
Supplement: sj-docx-1-mdm-10.1177_0272989X231208448 – Supplemental material for Potential Adverse Outcomes of Shared Decision Making about Palliative Cancer Treatment: A Secondary Analysis of a Randomized Trial [file sj-docx-1-mdm-10.1177_0272989X231208448.docx]

**APPENDIX A: Correlations and intraclass correlations of adverse outcomes**

Table A.1. Correlations between adverse outcomes

|  | Anxiety (STAI) | Loss of fighting spirit | Helplessness/  hopelessness | Tension (VAS) | Uncertainty |
| --- | --- | --- | --- | --- | --- |
| Anxiety (STAI) | 1 |  |  |  |  |
| Loss of fighting spirit | 0.27 | 1 |  |  |  |
| Helplessness/hopelessness | 0.59 | 0.54 | 1 |  |  |
| Tension (VAS) | 0.81 | 0.19 | 0.44 | 1 |  |
| Uncertainty | 0.42 | 0.2 | 0.31 | 0.33 | 1 |

Table A.2. Intraclass correlations of adverse outcomes

|  | ICC |
| --- | --- |
| Anxiety (STAI) | 0.007 |
| Loss of fighting spirit | -0.005 |
| Helplessness/hopelessness | -0.008 |
| Tension (VAS) | 0.017 |
| Uncertainty | 0.003 |

**APPENDIX B: Adverse outcomes’ spread around the mean**

**Table B1. Means and standard deviations of adverse outcomes at T0 (baseline), T1 (waiting room), T2 (one week) and T3 (three months)**

| **Outcome** | **T0 (baseline)**  Mean (SD) | **T1 (waiting room)** Mean (SD) | **T2 (one week)**  Mean (SD) | **T3 (three months)**  Mean (SD) |
| --- | --- | --- | --- | --- |
| **Anxiety (STAI)** (range 1-4) | 2.02 (0.57) | - | 1.97 (0.63) | 1.95 (0.54) |
| **Loss of fighting spirit** (range 4-16) | 10.82 (2.49) | - | 9.19 (2.49) | 10.75 (2.85) |
| **Helplessness/hopelessness** (range 6-24) | 11.74 (3.04) | - | 11.66 (3.07) | 11.40 (3.20) |
| **Tension (VAS)** (range 0-100) | - | 44.30 (28.76) | 27.74 (24.20) | 26.17 (24.55) |
| **Uncertainty** (range 0-100) | - | - | 28.43 (19.63) | - |
